# The Distribution of Activation Markers and Selectins on Peripheral T Lymphocytes in Preeclampsia

**DOI:** 10.1155/2017/8045161

**Published:** 2017-05-07

**Authors:** Anna Bajnok, Maria Ivanova, János Rigó, Gergely Toldi

**Affiliations:** ^1^First Department of Obstetrics and Gynecology, Semmelweis University, Budapest, Hungary; ^2^Department of Pediatrics, Medical Institute, Peoples' Friendship University of Russia, Moscow, Russia

## Abstract

**Introduction:**

Impaired maternal immune tolerance resulting in systemic inflammation plays a pivotal role in the pathogenesis of preeclampsia. Phenotypical changes of monocytes and neutrophil granulocytes have already been studied in preeclampsia, and some studies also included T lymphocyte activation markers; however, the results are controversial and a comprehensive analysis of activation markers is lacking. The characteristics of cellular adhesion molecules in preeclampsia are yet to be described.

**Material and Methods:**

Peripheral blood samples of 18 preeclamptic patients and 20 healthy pregnant women in the third trimester were evaluated using flow cytometry to characterize the cell surface expression of T lymphocyte activation markers and selectins.

**Results:**

We found an elevated ratio of HLA-DR and CD122-, CD62E-, and CD62L-expressing cells among the CD4+ T lymphocytes in PE in comparison to healthy pregnancy. No alterations were found in the prevalence of CD69-, CD25-, and CD62P-expressing lymphocytes and CD11c-expressing monocytes.

**Conclusions:**

Our findings support the role of activated T lymphocytes and specific cell adhesion molecules in the pathogenesis of preeclampsia.

## 1. Introduction

Of the many challenges that the immune system must face, the one presented by pregnancy is among the most complex and critically important ones. The development of specific immunotolerance towards the semiallogeneic fetal antigens is the most essential requirement for a successful pregnancy. The impairment of the maternal-fetal tolerance is the cornerstone of many pregnancy-specific diseases of which preeclampsia is linked to the highest degrees of maternal and fetal morbidity and mortality in developed countries. Preeclampsia (PE) occurs in approximately 5% of all pregnancies but is responsible for approximately 15% of all maternal mortality and 20% of premature births. The only causal therapy is acute iatrogenic delivery, which can lead to further perinatal complications. While several aspects of the pathogenesis are poorly understood, most findings support the central role of immune maladaptation-driven superficial placentation, leading to a systemic maternal inflammatory response, in which the role of activated T lymphocytes appears to be pivotal [1–3]. Monitoring the activation-induced responses of T lymphocytes could help gain a better understanding of the underlying pathophysiological processes.

Upon T cell activation, several cell surface markers are upregulated, each at a different stage of the activation process. The earliest activation marker is CD69, which is an inducible cell surface glycoprotein expressed upon activation via the TCR or the IL-2 receptor (CD25). It plays a role in the proliferation and survival of activated T lymphocytes [4, 5]. It is expressed at very low basal levels in resting lymphocytes; however, upon activation with phytohemagglutinin (a mitogen causing activation via the TCR) its expression increases in a time-dependent manner between 3 and 12 hours, remaining elevated until 24 hours and declining thereafter [6].

IL-2 plays a key role in the activation, survival, expansion, and function of T lymphocytes. It also appears to be crucial in the maintenance of regulatory T cell- (Treg-) mediated immunotolerance toward self-antigens, supported by the fact that IL-2-deficient mice develop severe autoimmunity with very low Treg and very high effector T numbers [7–9]. CD25 is the alpha chain of the trimeric IL-2 receptor and considered to be the most prominent cellular activation marker. It is expressed constitutively on the surface of several subsets of peripheral blood lymphocytes, such as regulatory and resting memory T cells. It is upregulated within 24 hours of stimulation of the TCR/CD3 complex and remains elevated for a few days [6, 10]. It plays a key role in responsiveness to IL-2 resulting in lymphocyte activation and further IL-2 production. Several cytokines released by monocytes and macrophages as well as other agents triggering T cell activation (such as oxidized LDL) are capable of inducing CD25 expression [11, 12]. CD122 is the *β*-chain of the IL-2 receptor, and upon expression, it further increases sensitivity of activated CD25+ T lymphocytes to IL-2. Together with the common *γ*-chain and CD25, it forms the high-affinity trimeric IL-2 receptor [13].

HLA-DR is a human class II major histocompatibility complex (MHC) antigen which is constitutively expressed on the surface of B lymphocytes, monocytes, and macrophages and appears at the late stages of activation on T and NK cells; thus, it is considered to be a very late activation marker [14].

Reddy et al. demonstrated that resting peripheral blood lymphocytes of healthy individuals show little or no expression of CD69 (very early) and moderate basal expression of CD25 (late) and HLA-DR (very late) markers. They demonstrated that the peak elevation of CD69 precedes the appearance of CD25 and HLA-DR, which showed progressive increase in expression after 24 hours [6].

Although the role of activated T lymphocytes is clear in the pathogenesis of preeclampsia, previous studies have been unable to detect alterations in the ratio of CD3/CD69 cells and the rate of CD25-expressing lymphocytes in preeclampsia in comparison with healthy pregnancy [2, 15].

Cell adhesion molecules are membrane proteins that regulate the adhesion of activated T cells to activated endothelial cells, thus are thought to play an important role in the regulation of inflammatory processes [16–18]. Selectins are a subset of cell adhesion molecules, which mediate the first step of adhesion (rolling) by decreasing leukocyte velocity upon endothelial contact [19, 20], which is followed by leukocyte arrest and endothelial transmigration. Naive T cells use CD62L (L-selectin) among other receptors to recognize and extravasate through specialized high endothelial venules of lymphoid tissue but lack the receptors to exit via other blood vessel types [21, 22]. The majority of activated T cells rapidly shed CD62L receptors and gain a new set of selectins which allow them to migrate to nonlymphoid tissues. Activated T cells are known to enter the skin via inflamed blood vessels, by binding via selectin ligands to CD62P (P-selectin) and CD62E (E-selectin) expressed by endothelial cells [23]. Until recently, studies have failed to detect the expression of E selectin on activated lymphocytes; thus, they were thought to be solely expressed by endothelial cells. However, Vainer et al. were able to demonstrate that in ulcerative colitis, mononuclear cells of the lamina propria are also E-selectin positive, indicating the altered role of this selectin in an ongoing inflammation [24].

*β*2 integrins (CD11a, CD11b, and CD11c) expressed by neutrophils and monocytes establish a firm leukocyte-endothelial adhesion by reducing rolling velocity of activated monocytes.

The aim of the present study was to gain a comprehensive understanding of the alterations of T lymphocyte activation markers and selectins in preeclampsia. We aimed to characterize the cell surface expression of lymphocyte activation markers CD69, HLA-DR, CD25, and CD122 and the expression of the selectins playing a role in the adhesion and extravasation of activated T lymphocytes CD62E, CD62L, and CD62P. We also aimed to characterize the CD11c expression of monocytes to monitor their activation rate.

## 2. Material and Methods

### 2.1. Sample Collection

We collected peripheral venous blood samples from 18 preeclamptic pregnant women on average at the 36th week of pregnancy. PE was diagnosed according to the standard, internationally accepted criteria, which include hypertension (defined as systolic blood pressure and/or diastolic blood pressure ≥ 140 mmHg and ≥90 mmHg, resp.) occurring after 20 weeks of gestation and proteinuria (defined as presence of ≥0.3 g protein in a 24-hour urine specimen). 20 healthy pregnant (HP) women were enrolled as controls on average at the 36th gestational age. Clinical characteristics of participants are summarized in Table 1. PE was regarded as severe if any of the following criteria was present: blood pressure ≥ 160 mmHg systolic or ≥110 mmHg diastolic or proteinuria ≥5 g/24 h (or 3+ on dipstick). Early onset of PE was defined as onset of the disease before 34 weeks of gestation. Our study was reviewed and approved by an independent ethical committee of the institution (Scientific and Research Ethics Committee, Semmelweis University, Budapest, Hungary; date of issue and registration number: 2008.04.08/ TUKEB 56/2008), and written informed consent was obtained from all participants. The study was adhered to the tenets of the most recent revision of the Declaration of Helsinki.

### 2.2 Flow Cytometry

9 ml of peripheral venous blood was collected from each woman in lithium heparin-treated tubes (BD Vacutainer, BD Biosciences, San Jose, CA). Peripheral blood mononuclear cells (PBMCs) were isolated by standard density gradient centrifugation (Ficoll Paque, Amersham Biosciences AB, Uppsala, Sweden; 25 min, 400 g, room temperature). Cells were kept at −80°C in Fetal Bovine Serum containing 10% DMSO until analysis. After thawing, cells were washed twice in phosphate-buffered saline.

PBMCs were incubated for 30 min at room temperature in the dark with the following antibodies: APC-Cy7-conjugated CD4, PerCP-conjugated HLA-DR, APC-conjugated CD62E, PE-Cy7-conjugated CD62L, PE-conjugated CD62P, FITC-conjugated CD25, PE-Cy7-conjugated CD11c, PE-conjugated CD122, and APC-conjugated CD69 (BD Biosciences). After washing, fluorescent data were registered on a BD FACSAria flow cytometer (BD Biosciences). Data were processed using the FACSDiVa software. 200000 cells per sample were recorded.

### 2.3. Statistics

Data are expressed as median and interquartile range (IQR). Comparisons between the two patient groups were made with the Mann–Whitney *U* test, as a test of normality (according to Kolmogorov-Smirnoff) indicated nonnormal distribution of data. *p* values less than 0.05 were considered significant. Sample size was estimated to achieve 80% power with 0.6 effect size to detect differences between the two study populations. Statistics were calculated using the GraphPad software (Prism version 5.00 for Windows, GraphPad Software, San Diego, CA, USA).

## 3. Results

### 3.1. Activation Markers

**Figure 1:**
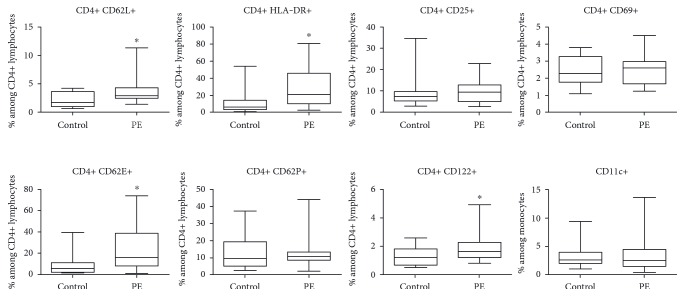
Box plots representing the frequency of the investigated cell subsets in healthy pregnancy (control) and preeclampsia (PE). Horizontal line: median; box: interquartile range (25–75 percentile); whisker: range. ^∗^*p* < 0.05 versus control.

Our results are summarized in Figure 1 and Table 2. Within CD4+ T lymphocytes, we found no alterations in the prevalence of CD69-expressing T cells (very early activation marker) and CD25-expressing T cells (late activation marker). However, the prevalence of CD4+ HLA-DR+ T lymphocytes was significantly higher in women with PE, than in HP women (6.19% (3.02–13.73) versus 21.05% (9.98–46.03), *p* = 0.0075).

In PE, within the CD4+ T cells, the percentage of activated CD122+ lymphocytes was also higher (1.20% (0.67–1.81) versus 1.63% (1.20–2.28), *p* = 0.0437). We found no alterations in the prevalence of CD11c+ monocytes in PE.

### 3.2. Selectins

We found an elevated prevalence of CD4+ CD62L+ lymphocytes in PE (1.74% (1.03–3.64) versus 2.91% (2.49–4.30), *p* = 0.0124). We found that CD4+ lymphocytes isolated from both groups expressed CD62E, with a distinct elevation in the prevalence of CD4+ CD62E+ lymphocytes in women with PE compared to HP women (5.77% (2.26–10.96) versus 16.10% (8.25–38.80), *p* = 0.0141). The prevalence of CD62P+ lymphocytes in the CD4+ T cell population did not differ between the two groups.

## 4. Discussion

In this study, we analyzed the expression of activation markers and selectins on the surface of peripheral T lymphocytes and monocytes in preeclampsia compared to healthy pregnancy.

The role of activated T lymphocytes in the pathogenesis of PE is supported by several studies [25, 26]. Some also described activation markers; however, the results are controversial and a comprehensive analysis of activation markers is lacking. While some studies observed elevated levels of CD25+ T lymphocytes [27], others reported decreased percentage of CD3+ cells and CD25+ T lymphocytes with increased prevalence of CD4+ HLA-DR+ T lymphocytes in PE compared with HP [28]. Therefore, we aimed to complete a comprehensive analysis of the most important T lymphocyte activation markers.

Wilczyński et al. isolated lymphocytes from the third trimester decidua of preeclamptic pregnant patients and healthy pregnant controls and investigated the expression of certain cell surface receptors, including CD69; however, they found no alterations in the CD3/CD69 decidual lymphocyte subset in preeclampsia compared to healthy pregnancy [15]. Rieger et al. investigated the changes of decidual leukocyte populations in preeclampsia; however, they were unable to detect any alterations in the rate of CD25-expressing cells in preeclampsia compared to healthy pregnancy [2]. Our results are in line with these previous findings, since we found no difference between preeclamptic and healthy pregnant women in the expression rate of very early activation marker CD69 and late activation marker CD25. However, we found a significant elevation in the rate of HLA-DR-expressing cells among the CD4+ T lymphocytes in PE compared to HP. HLA-DR is the very late activation marker of T lymphocytes, the expression of which starts elevating after 24 hours following a stimulus and remains high for up to a few weeks. Preeclampsia is a chronic, often slowly developing, progressive disease, which results in a chronic inflammatory status accompanied by increased antigen presentation. Therefore, it is tempting to hypothesize that since at the time of sampling, there has been an ongoing inflammation for up to a few weeks; the very early (CD69) and even the late (CD25) activation markers are no longer significantly elevated on the surface of T lymphocytes. However, it appears that the very late activation marker (HLA-DR) closely mirrors the chronic T cell activation present in PE. Results from previous studies are somewhat incongruent, since some found no alterations in the prevalence of CD4+ CD25+ lymphocytes [29], while others found an elevated level of CD25 expression among CD4+ lymphocytes [25, 27]. Similarly, some studies reported no alterations in the expression of HLA-DR among CD4+ lymphocytes [25], while others found an elevated ratio of CD4+ HLA-DR+ lymphocytes [28]. It is important to note that it is almost impossible to predict the time which has passed from the development of PE until the time of clinical admission; thus, it is highly challenging to standardize the time of sample collection. Since, at different points of lymphocyte activation process, different activation markers are upregulated, it is entirely possible that the differences in the time passed between the onset of PE and the sample collection could account for the abovementioned incongruity, especially since we also observed an elevating tendency in the expression of CD25 in CD4+ lymphocytes, which did not reach a significant level.

Loewendorf et al. investigated the ratio of T cells at the uteroplacental interface (UPI) and in the peripheral blood in healthy pregnancy and found that the distribution of CD4 and CD8 T cell populations in the peripheral blood largely mirror that in the UPI. They applied a simple staining and gating strategy developed by Sakaguchi [30] that allows for the analysis of three regulatory T cell (Treg) subtypes with the following distinct biological features: resting Tregs (FoxP3 low, CD45RA+), cytokine Tregs (FoxP3 low, CD45RA-), and activated Tregs (FoxP3 high, CD45RA+). The HLA-DR expression of the three Treg subsets was described, since HLA-DR expression identifies a highly suppressive Treg population. They found that while resting Treg cells express low levels of HLA-DR, there is a focal enrichment of HLA-DR-expressing cytokine Tregs. This increase in HLA-DR+ cytokine Tregs at the UPI mirrored the increase of the HLA-DR+ CD8 T cell subset, resulting in a similar ratio of responder CD8 T cells and suppressor regulatory T cells at the UPI and at the periphery. They hypothesized that the alterations of the ratio of HLA-DR-expressing T cell subsets might be important in pathological conditions, such as preeclampsia [1].

CD122, which is the *β*-chain of the IL-2 receptor, is an important T cell activation antigen; however, it has not been previously investigated in preeclampsia. We found an elevated rate of CD122-expressing cells among the CD4+ T lymphocyte population in PE compared with HP. As a component of the high-affinity trimeric IL-2 receptor, CD122 plays a key role in increasing the sensitivity to IL-2, thus regulating T lymphocyte survival, expansion, and activation. The elevated expression of this cell surface antigen indicates an increased activation rate among the CD4+ lymphocytes. These findings further support the role of activated T lymphocytes in the development of PE.

CD62E, CD62L, and CD62P play a central role in the adhesion of activated T lymphocytes to activated endothelial cells. Rolling enables leukocytes to sense proinflammatory cytokines and chemokines produced by activated endothelial cells at the site of inflammation [31, 32], and thus selectins also regulate the homing of activated T lymphocytes to the site of inflammation. Therefore, lymphocytes are able to exit to lymphoid tissue for maturation (via CD62L) and to the site of inflammation in nonlymphoid tissues (connections via CD62E and CD62P). Until recently, CD62E was believed to be expressed solely by endothelial cells; however, Vainer et al. demonstrated that in colonic biopsies obtained from ulcerative colitis patients in the active stage of the disease, E-selectin is expressed not only by epithelial and endothelial cells of the luminal membrane but also by mononuclear cells [24]. E-selectin expression is also modified by a number of cytokines. Proinflammatory cytokines, such as TNF-*α* and IL-1, upregulate the transcription of CD62E, while TGF-*β* downregulates the transcription of CD62E [33, 34]. Harashima et al. demonstrated that E-selectin expression can be induced not only on endothelial cells but also on CD41+ T cells and on Jurkat cells by TNF-*α* [35]. It is likely that in certain inflammatory conditions, in order to enhance the adhesion process, E-selectin expression is not limited to endothelial cells but is also expressed by mononuclear cells. Our results support this hypothesis, since we found a threefold elevation in the rate of CD62E-expressing CD4+ T lymphocytes in PE compared with HP. It is important to note that CD4+ T lymphocytes also expressed CD62E in HP women, suggesting that pregnancy alone could alter the expression of this selectin. However, the significant elevation found in PE indicates that it could play an important role in the increased extravasation of activated T lymphocytes in preeclampsia.

We also found an elevated rate of CD62L-expressing cells among CD4+ T lymphocytes, which indicates an increased tendency of naive T lymphocytes to exit to lymphoid tissue, where they meet specific antigens and undergo maturation and activation process and rapidly shed CD62L thereafter. CD62L is also considered a late T lymphocyte activation marker, since naive T lymphocytes are characterized by high expression of CD62L (CD62hi), whereas activated T lymphocytes can be characterized by low expressions of CD62L (CD62lo), and the rate of CD62L shedding can be utilized to describe leukocyte activation rate [36]. The prevalence of CD62P+ T lymphocytes did not differ between the two study groups; therefore, we hypothesize that the role of this selectin is secondary in the pathophysiology of PE.

Activated monocytes play a role in antigen presentation and the activation process of T lymphocytes. An elevated rate of monocyte activation could indicate the presence of increased antigen presentation. *β*2 integrins (CD11a, CD11b, and CD11c) play a role in the adhesion of activated monocytes to activated endothelial cells. Mellembakken et al. reported an elevated expression of CD11a and CD11c integrins on the surface of monocytes isolated from uterine venous blood samples compared with those isolated from antecubital venous samples in preeclampsia. They found an elevated expression of integrins on neutrophils and monocytes isolated from uterovenous samples in preeclampsia when compared with uncomplicated pregnancy. They hypothesized that there is an increased rate of monocyte activation taking place at the uteroplacental passage leading to enhanced leukocyte-endothelial interactions in preeclampsia, resulting in increased neutrophil infiltration and elevated presence of mononuclear perivascular cells in the decidua. They suggested that therapeutic blocking of integrin-mediated leukocyte-endothelial interaction might be beneficial [37]. We found no alteration in the rate of CD11c-expressing peripheral monocytes. A possible explanation is the aforementioned difference in the prevalence of integrin-expressing monocytes between uterovenous and antecubital venous samples, indicating the accumulation of activated monocytes at the UPI.

## 5. Conclusion

In summary, our current findings are in line with previous findings and suggest that activated T lymphocytes play an important role in the pathogenesis of PE [38]. While the very early (CD69) and late (CD25) T lymphocyte activation markers were no longer elevated, HLA-DR, the very late activation marker, and CD122, a pivotal regulator of T cell activation and survival, was significantly higher in preeclampsia than in healthy pregnancy, mirroring the chronic inflammatory process. From the cell adhesion molecules, CD62E, which is presumably only expressed by lymphocytes in inflammatory conditions, was significantly upregulated in PE compared to HP. However, it also appeared in the surface of lymphocytes isolated from healthy pregnant controls, indicating that pregnancy alone could be an altering factor in the expression of this selectin. The elevated expression of CD62L suggests the increased maturation and activation of naive T cell subsets. These cell adhesion molecules could play a role in the inflammatory site-specific extravasation of lymphocytes in PE.

## Figures and Tables

**Table 1:** Clinical characteristics of study participants.

	Healthy pregnant women (*n* = 20)	Preeclamptic women (*n* = 18)
Maternal age (years)	33.5 (30–35.25)	32.5 (26.5–34.75)
BMI	26.5 (23.4–28.3)	30 (28–33.4)
Systolic blood pressure (mmHg)	110 (104.75–116.25)	140^∗^ (138–155)
Diastolic blood pressure (mmHg)	67.5 (60–70)	90^∗^ (90–100)
Proteinuria (g/24 h) (cross)	—	0.91 (0.65–2.035) (+++)
Gestational age at diagnosis (week)	—	36 (28–38)
Gestational age at blood sample collection (week)	36 (34–37)	36 (29.25–38)
Fetal birth weight (g)	3180 (2985–3632.5)	2560^∗^ (1295–3450)
Early onset preeclampsia	—	7 (39%)
Severe preeclampsia	—	12 (67%)

Data are presented as median (interquartile range) for continuous variables and as number (percentage) for categorical variables. ^∗^*p* < 0.05 versus healthy pregnant women.

**Table 2:** Prevalence of the investigated cell subsets.

	Healthy pregnancy (*n* = 20)	Preeclampsia (*n* = 18)	*p* value
Activation markers			
CD4+ CD69+/CD4+	2.28 (1.77–3.27)	2.61 (1.68–2.97)	0.8151
CD4+ CD25+/CD4+	7.38 (5.39–9.64)	9.51 (5.03–12.70)	0.5686
CD4+ HLA-DR+/CD4+	6.19 (3.02–13.73)	21.05^∗^ (9.98–46.03)	0.0075
CD4+ CD122+/CD4+	1.20 (0.67–1.81)	1.63^∗^ (1.20–2.28)	0.0437
CD11c+/monocytes	2.57 (1.94–3.83)	2.43 (1.39–4.39)	0.5827
Selectins			
CD4+ CD62E+/CD4+	5.77 (2.26–10.96)	16.10^∗^ (8.25–38.80)	0.0141
CD4+ CD62L+/CD4+	1.74 (1.03–3.64)	2.91^∗^ (2.49–4.30)	0.0124
CD4+ CD62P+/CD4+	9.53 (5.04–19.05)	10.70 (8.54–13.20)	0.6068

Data are presented as median (interquartile range). ^∗^*p* < 0.05 versus healthy pregnant women.
